# Emotional intelligence and stress and their relationship with breastfeeding self-efficacy in mothers of premature infants

**DOI:** 10.1186/s12905-023-02849-4

**Published:** 2024-01-03

**Authors:** Mohammad Arshadi Bostanabad, Mina Hosseinzadeh, Zahra Molazemi, Hossein Namdar Areshtanab

**Affiliations:** 1 Department of Pediatric Nursing, Nursing and Midwifery Faculty, Tabriz University of Medical Sciences, Tabriz, Iran; 2 Department of Community Health Nursing, Nursing and Midwifery Faculty, Tabriz University of Medical Sciences, Tabriz, Iran; 3 Department of Mental Health and Psychiatric Nursing, Nursing and Midwifery Faculty, Tabriz University of Medical Sciences, Tabriz, Iran

**Keywords:** Emotional intelligence, Stress, Self-efficacy, Premature infant, Neonatal intensive care unit

## Abstract

**Background:**

Premature infants need to be hospitalized in the neonatal intensive care unit (NICU) for long periods of time, which can increase anxiety and stress in their mothers. Additionally, the breastfeeding rate is lower among preterm infants. This study aimed to determine stress levels and emotional intelligence in mothers of preterm infants and their relationship with breastfeeding self-efficacy.

**Methods:**

This descriptive-correlational study was performed with a convenience sampling of 210 mothers of premature infants admitted to the neonatal intensive care unit in Tabriz, Iran in 2021. Data collection tools included socio-demographic checklist, perceived stress scale (PSS14), Dennis’ breastfeeding self-efficacy scale, and the Schering emotional intelligence questionnaire. Data were analyzed using SPSS software version 16 via descriptive and inferential statistics (Pearson correlation and one-way ANOVA and modified general linear model).

**Results:**

Study findings demonstrated that most of the mothers had low stress (75.2%) and high breastfeeding self-efficacy (61.9%). The mean (SD) of emotional intelligence of the participants was 88.18 (16.60), ranging from 33 to 165. The results of the general linear model by modifying the demographic characteristics showed that the variables of emotional intelligence (B = 0.23, *P* = 0.03), stress (B=-0.56, *P* = 0.01), gestational age (B = 2.81, *P* < 0.001) and number of deliveries (B = 9.41, *P* < 0.001) were predictors of breastfeeding self-efficacy.

**Conclusion:**

The findings showed that mothers of preterm infants had low emotional intelligence, and the majority of them had low perceived stress and high breastfeeding self-efficacy. Findings highlight the importance of addressing maternal stress and enhancing emotional intelligence to promote successful breastfeeding in mothers of preterm infants. Healthcare providers and managers are encouraged to offer support and educational programs to mothers of preterm infants, aiming to enhance their emotional intelligence. Further research and interventions focusing on these factors are warranted to improve the overall well-being of both mothers and infants in the neonatal intensive care unit.

## Introduction

Premature infants are defined as infants who are born at less than 37 weeks of gestation [1]. Approximately 15 million premature infants are born worldwide each year [2]. Iran is one region with a high prevalence of preterm births accounting for approximately 5.2–7.3% of the total number of newborn infants [3]. Premature infants need to be hospitalized in the neonatal intensive care unit (NICU) for long periods of time, which increases the anxiety and stress of parents [4]. Many critical situations, such as respiratory distress syndrome, intraventricular hemorrhage, hypoglycemia, and neurological defects, threaten infants’ lives and can cause a high amount of stress in parents [5, 6]. It has been reported that mothers of preterm infants admitted to the NICU experience high levels of mental health problems [7].

Emotional intelligence (EI) skills are potentially effective in coping with stress. In other words, emotional intelligence helps people function through stress management and emotion control [8]. In a recent study, an association between emotional intelligence and using problem-focused coping strategies was found [9]. It has been reported that emotional intelligence is associated with self-efficacy and better performance in the areas of self-regulation, assertiveness, independence, empathy with others, control, and optimism [10].

Successful breastfeeding is one of the important roles of a mother [11]. It is an important part of care because it reduces the risk and severity of possible preventable complications of prematurity during and after hospitalization in the neonatal intensive care unit [12]. However, mothers who have a premature infant are less likely to have successful breastfeeding than mothers of term infants [3]. Although it depends on different factors, such as the attitude toward breastfeeding, the availability of breastfeeding facilities, and the support of those around the mother [13], the separation of the mother from the infant who needs to be hospitalized in the intensive care unit [14], poor sucking of premature infants [15] and mental and emotional problems caused by preterm delivery in these mothers [16].

Self-efficacy means a person’s belief in their ability to perform a particular task or behavior [17], and breastfeeding self-efficacy is a valuable framework of Bandura’s social cognitive theory that predicts maternal breastfeeding behavior, degree of self-confidence and her ability to breastfeed [18, 19]. While the early postpartum period plays a pivotal role in shaping breastfeeding self-efficacy through individual-social factors [20, 21], limited research has explored the intricate link between emotional intelligence and breastfeeding self-efficacy [22]. This knowledge gap underscores the need for further investigation into the nuanced interplay between emotional intelligence and breastfeeding self-efficacy. Given the potential ramifications of preterm labor on maternal mental health and its subsequent influence on breastfeeding success, our study aims to elucidate the relationship between stress, emotional intelligence, and breastfeeding self-efficacy, recognizing emotional intelligence as a potential tool for stress management in this context.

## Method and materials

In this cross-sectional descriptive study, 210 mothers of premature infants admitted to the neonatal intensive care unit of Al-Zahra, Pediatrics and Taleghani hospitals, which are educational hospitals of Tabriz University of Medical Science, participated. The sample size based on the study of Karakoc et al. and considering (α = 0.05, d = 3% of mean and 95% confidence interval) [22] by using the sample size formula was determined to be 146, 206 and 143 participants, respectively. We selected the highest sample size, which was 206, and by considering a 10% probability attrition rate, the final sample was 235.

Inclusion criteria in this study were hospitalization of the infant in the neonatal intensive care unit, mothers who were allowed to breastfeed, having literacy of reading and writing and being breastfeed. Exclusion criteria were congenital anomalies in the infant, any domestic violence against the mother, history of any known physical and mental illnesses, history of infertility, experiences of stressful events according to the person’s self-report and having a nonresponse rate of more than 25% on the questionnaire.

### Sampling

Mothers were selected by a convenience sampling method among the mothers of preterm infants hospitalized in NICUs of three selected hospitals according to inclusion and exclusion criteria from March to September 2021. We collected the data using paper questionnaires, which were distributed in person to the participants. Each participant received a physical copy of the questionnaire and was asked to complete and return it. Among the participants, 25 questionnaires had more than 25% missing items, and 210 subjects entered the analysis of data.

### Data collection tool

Data collection was performed by 4 questionnaires: a socio-demographic questionnaire, perceived stress questionnaire, Denis breastfeeding self-efficacy questionnaire and Schering emotional intelligence questionnaire.

The socio-demographic questionnaire had 21 items about parents’ and infants’ characteristics. The parents’ characteristics included age, duration of the marriage, education, age and education of the spouse, job of the spouse, family income level, place of residence, maternal drug use, stress during the past month, congenital anomalies in the baby, history of known physical and mental illnesses in the mother, number of pregnancies, number of deliveries, history of breastfeeding, breastfeeding duration, type of delivery, history of infertility and gestational age. Information about the infant’s characteristics included sex, age, height, weight, diagnosis and length of hospital stay.

Perceived Stress Questionnaire: The PSS-14 perceived stress questionnaire was used to measure perceived stress at birth. This questionnaire was designed and used by Cohen et al. in 1983 [23]. The design of this self-completion questionnaire is based on a 5-point Likert scale from never = 0, almost never = 1, sometimes = 2, often = 3 to most of the time = 4 points. Items 4-5-6-7-9-10 and 13 are scored in reverse (never = 4 to most of the time = 0). The score range was between 0 and 56, and a higher score indicates higher perceived stress. The cutoff point of the scale was 28, and a score lower than 28 indicated low perceived stress, and a score higher than 28 indicated high perceived stress. The reliability of the Persian version of this questionnaire was calculated by Bastani et al. using the internal consistency method and was reported to be 0.74 [24].

Denis Breastfeeding Self-Efficacy Questionnaire: This self-report questionnaire has 33 questions and a 5-point Likert scale from strongly agree (score 5) to strongly disagree (score 1). Scores range from 33 (lowest level) to 165 (highest level). A score of 33–76 is considered low self-efficacy, a score of 77–120 is considered moderate self-efficacy, and a score of 121–165 is considered high self-efficacy. This tool was designed by Bandura in 1997 and was first used by Fax and Dennis in breastfeeding [25]. The validity and reliability of this questionnaire in Iran were evaluated by Hassanpour et al., and the Cronbach’s alpha coefficient was calculated to be 0.82 [26].

Schering Emotional Intelligence Questionnaire: It was developed in 1996 based on Goleman’s theory (1995) [27] and was preliminarily standardized for postgraduate students of Allameh Tabatabai University by Mansouri. The main form had 70 questions and was standardized to 33 questions. The grading is performed on a 5-point scale from 1 to 5. Each individual receives 6 separate scores, 5 of which are related to each component (spontaneity 7 questions, self-awareness 8 questions, self-control 7 Questions, social awareness or empathy 6 questions, social skills 5 questions) and 1 score in general. The score ranges from 33 to 165. The Cronbach’s alpha coefficient was calculated to be 0.85 in Monsouri et al.’s study [28]. To assess the face validity of the questionnaires, we conducted qualitative methods by piloting the questionnaires among ten professors at Tabriz University of Medical Sciences. Based on their feedback, necessary corrections were made to improve the questionnaires.

### Analysis

Statistical analysis was performed using SPSS software version 16. The normality of quantitative data was assessed using skewness and kurtosis, which had a normal distribution. Descriptive statistics of frequency (percentage) and mean (standard deviation) were used to describe socio-demographic characteristics. Pearson correlation, independent *t*, and one-way ANOVA tests were used to determine the relationship of breastfeeding self-efficacy with emotional intelligence and stress and other socio-demographic variables in bivariate analysis. In the next step, variables significantly related to breastfeeding self-efficacy (*P* < 0.2) (potential confounding variables), stress and emotional intelligence (independent variables), and breastfeeding self-efficacy (dependent variable) were entered into the general linear model.

## Results

A total of 210 mothers of preterm infants who were hospitalized in the NICU were included in our study. The mean (SD) age of mothers was 28.28 (6.16) years, and the mean age of infants was 19.08 (17.88) days (age range: 2–90 days). Other socio-demographic characteristics of the participants are shown in Table 1.

**Table 1:** Socio-demographic characteristics of participants and their association with breastfeeding self-efficacy (N = 210)

	Variable		Mean (SD)*					*P* value
**Infant** **Mother**	Age (days)		19.08(17.88)					r=-0.28, *P* < 0.001
	Hospitalization length		19.74 (18.21)					r = 0.32, *P* < 0.001
	Age (year)		28.28(6.16)					r=-0.34, *P* = 0.34
	Duration of marriage		5.92(4.48)					r = 0.11, *P* = 0.25
	Number of pregnancies		1.80(0.80)					r = 0.14, *P* = 0.01
	Number of deliveries		1.50(0.58)					r = 0.33, *P* < 0.001
	Gestational age (Weeks)		33.68 (2.84)					r = 0.43, *P* < 0.001
**Education level**	High school or less		150(71.4), t = 1.80, *P* = 0.09					
	University degree		60(28.6)					
**Job**	Housekeeper		194(92.8), t = 1.18, *P* = 0.24					
	Employed		15(7.2)					
**Education level of spouse**	High school or less		148(70.5), t=-1.04, *P* = 0.40					
	University degree		62(29.5)					
**Job of spouse**	Unemployed		12(5.7), t=-1.23, *P* = 0.22					
	Employee		198(94.3)					
**Income**	Sufficient		77(36.7), F = 0.13, *P* = 0.26					
	Fairly sufficient		83(39.5)					
	Insufficient		50(23.8)					
**Living place**	Urban		162(77.5), t=-1.92, *P* = 0.06					
	Rural		47(22.5)					
**Previous Breastfeeding experiences**	Yes		97(48.7), t = 3.32, *P* = 0.001					
	No		102(51.3)					
**Breastfeeding education**	Yes		62(34.8), t = 1.34, *P* = 0.78					
	No		116(65.2)					
**Type of delivery**	Vaginal		73(34.8), t = 2.01, *P* = 0.45					
	Cesarean		137(65.2)					

Note: SD* = Standard Deviation

The mean (SD) of perceived stress and breastfeeding self-efficacy was 23.31 (7.63) and 122.32 (20.22) respectively, and the majority of participants 158 (75.2%) had low stress and 130 (61.9%) had high self-efficacy. The mean (SD) of emotional intelligence of the participants was 88.18 (16.60), and the dimension of self-control had the highest mean of 20.13 (5.47). (Table 2).

**Table 2:** Mean and standard deviation of self-efficacy, perceived stress, and emotional intelligence in mothers of preterm infants (N = 210)

Variable		N (%)		M(SD)
**Stress**	Low	158(75.2)		23.31(7.63)
	High	52(24.8)		
**Breastfeeding self-efficacy**	Low	4(1.9)		122.32(20.11)
	Medium	76(36.2)		
	High	130(61.9)		
**Emotional intelligence**	Self-control	Min	Max	M(SD)
		8	33	20.13(5.47)
	Self-motivation	11	26	18.88(3.76)
	social skills	7	23	13.43(3.25)
	Social awareness	7	25	15.63(4.14)
	Self-awareness	7	26	16.59(4.12)
	Total	44	127	88.18(16.60)

There was a negative significant relationship between breastfeeding self-efficacy and stress (r=-0.16, *P* = 0.004) and a positive non-significant relationship with emotional intelligence (r = 0.08, *P* = 0.14). (Table 3). Among the socio-demographic factors, the results of bivariate tests showed a significant relationship between the breastfeeding self-efficacy score and the variables of infant age (*P* < 0.001), hospitalization length (*P* < 0.001), number of pregnancies (*P* = 0.01), number of deliveries (*P* < 0.001), gestational age (*P* < 0.001) and experiences of previous breastfeeding (*P* = 0.001) (Table 1). The variables that showed a statistically significant relationship with breastfeeding self-efficacy (*P* < 0.2) were adjusted as independent variables. The adjusted variables were entered into the general linear model. The findings showed that the number of deliveries, gestational age, emotional intelligence and stress were statistically significant predictors of breastfeeding self-efficacy. Higher breastfeeding self-efficacy was observed in mothers who had a greater number of delivers (B = 9.41, *P* < 0.001), higher gestational age (B = 2.81, *P* < 0.001), higher score of emotional intelligence (B = 0.23, *P* = 0.03) and lower perceived stress (B=-0.56, *P* = 0.01). (Table 4)

**Table 3:** *The association between emotional intelligence and perceived stress and self-efficacy in mothers of premature infants (N = 210)*

Variable	Stress		emotional intelligence			
	R	*p*		R	P	
**Breastfeeding Self-efficacy**	-0. 16	0.004		0. 085	0.146	

**Table 4:** *Predictor factors of breastfeeding self-efficacy based on the general linear model* (N = 210)

Variable	B (95% Confidence Interval)	*P* value
**Breastfeeding Experience (Reference: No)**		
**Yes**	-0.68 (-5.78 to 4.65)	0.98
**Education (Reference: University)**		
**Diploma or less**	3.65 (-2.40 to 9.37)	2.34
**Living location (Reference: Village)**		
**City**	-0.47 (-7.58 to 3.56)	0.565
**Infant age**	0.27 (-0.64 to 0.18)	0.057
**Marriage duration**	0.28 (-0.31 to 0.88)	0.34
**Hospitalization length**	-0.157 (-0.56 to 0.24)	0.24
**Number of pregnancies**	1.64 (0.43 to 2.84)	0.65
**Number of deliveries**	9.41 (4.82 to 14.71)	< 0.001
**Gestational age**	2.81 (2.96 to 3.62)	< 0.001
**Total emotional intelligence score**	0.234 (0.02 to 0.44)	0.03
**Total stress score**	-0.56(-1.07 to -0.10)	0.01

## Discussion

The present study aimed to determine breastfeeding self-efficacy (BSE) and its relationship with perceived stress (PS) and emotional intelligence (EI) among mothers of preterm infants. The findings showed that the majority of participants had low perceived stress and high breastfeeding self-efficacy. The number of deliveries, gestational age, emotional intelligence and stress were statistically significant predictors of breastfeeding self-efficacy.

Our findings showed that 75.2% of the mothers of preterm infants had low stress and 24.8% had high stress. In a similar study by Ong (2018), more than half of the mothers of preterm infants reported a moderate level of stress [29]. In another recent study, the level of stress in mothers with infants hospitalized in the NICU was high [30]. Alteration in the parenting role was reported as the major cause of stress in mothers of preterm infants [29]. The stress of mothers of preterm infants is probably rooted in her uncertainty about the health status of the premature infant and how to care for the infant in this situation [31]. In the present study, the majority of mothers reported a low level of perceived stress. We performed the study when the infants’ situations were stable and mothers could breastfeed them and because they had passed the critical situation of the infants, their stress level was decreased. Maybe the other probable cause of the low level of stress in the majority of mothers is the high collaboration of mothers in infant care, which may cause a decrease in stress and negative feelings.

Regarding EI, our findings showed that the mean (SD) of emotional intelligence was 88.18 (16.60) in the score range of 33–165, which shows a low level of emotional intelligence and needs to be improved. In the Fu et al. (2020) study, the mean (SD) of emotional intelligence in mothers with twin pregnancies was reported to be 74.71 (9.23) [32], which is similar to our findings. In similar studies, 57.1% of pregnant women [33] and 55% of mothers reported normal levels of EI during the postpartum period [34]. Among the dimensions of emotional intelligence, “self-control” has the highest mean in the present study. In Ebrahimi et al.’s study, the highest score among the dimensions of emotional intelligence in mothers was the self-awareness dimension [35]. In a similar longitudinal study, it was reported that the dimension of “self-control” increased after childbirth [36]. This improved self-control would then enable better emotion and stress management, which would allow new parents to better adapt to the new family structure and take care of the infant’s needs; it is also mentioned that the investment in parenthood may lead to increasing family expectations. Such expectations would lead to an increase in self-control, which may be considered a form of emotional growth [37].

In the present study, 61.9% of participants had a high level of BFSE. Similarly, a previous study reported that the mothers of preterm infants had low BFSE, which caused non-continuity in breastfeeding [19]. Breastfeeding self-efficacy is especially highlighted in mothers of preterm infants since they face additional obstacles leading to the delayed onset of breastfeeding [22]. In some other studies, mothers in the postpartum period achieved high scores in BFSE [38, 39]. Breastfeeding education during prenatal care, baby-friendly hospitals and encouraging mothers to breastfeed immediately after childbirth, cultural properties, and Islam’s emphasis on breastfeeding can be effective factors in this regard. The hospitals where the study was performed were baby-friendly hospitals, and mothers had unlimited permission to be at the bedside of their infants. These factors may have caused the high level of BFSE in our study.

The present study found a significant positive relationship between emotional intelligence and breastfeeding self-efficacy, as well as a negative relationship between stress and breastfeeding self-efficacy. However, a similar study by Karakoç et al. did not find a significant relationship between emotional intelligence and breastfeeding self-efficacy in early postpartum women [22]. The difference in sample characteristics between the two studies, with our study focusing on mothers with preterm infants, may explain these discrepancies. Nevertheless, further studies in this area are recommended, as there is a lack of research available.

Regarding the relationship between breastfeeding self-efficacy and stress, previous studies have also reported similar findings. For instance, a recent study conducted in Iran demonstrated that increasing the score of breastfeeding self-efficacy significantly decreases perceived stress [40]. The presence of stress and negative emotions can undermine a mother’s confidence and lead to self-doubt, anxiety, and sadness, making it more challenging for them to breastfeed effectively. Consequently, this can further lower their breastfeeding self-efficacy [41].

Furthermore, our findings indicated that breastfeeding self-efficacy increases with an increase in the number of deliveries. This aligns with a study conducted by Poorshaban et al., which found that mothers with more children tend to have higher breastfeeding self-efficacy [42]. However, another recent study did not find a relationship between breastfeeding self-efficacy and the number of pregnancies and deliveries [40]. It is plausible that an accumulation of pregnancy and childbirth experiences, particularly within the unique context of preterm births, contributes to mothers’ increased proficiency in breastfeeding, subsequently bolstering their self-efficacy. Furthermore, in concordance with our study focusing on mothers of preterm infants, those with prior breastfeeding experiences reported lower perceived stress and higher breastfeeding self-efficacy.

Our findings showed that mothers with higher gestational age had higher breastfeeding self-efficacy. The lower the gestational age, the higher the likelihood that a baby may encounter difficulties in latching onto the breast, maintaining an effective suck, or coordinating their breathing while breastfeeding. These challenges can adversely affect a mother’s breastfeeding self-efficacy, leading her to feel inadequate or attribute blame to herself for the difficulties [42].

### Limitations

One limitation inherent in our study pertains to the exclusive focus on evaluating breastfeeding self-efficacy, necessitating the inclusion of mothers with infants capable of consuming breast milk and maintaining a stable condition. This selective criterion might compromise the generalizability of our findings to a broader population of mothers with preterm infants facing different circumstances. Additionally, it is crucial to acknowledge the reliance on self-reported data in our study. While we assumed the accuracy of the participants’ responses, the intrinsic subjectivity of self-reporting introduces a potential limitation to the overall reliability of the gathered data. As such, caution should be exercised in interpreting the results, recognizing the inherent limitations associated with self-reported information.

## Conclusions

This research emphasizes the significance of addressing maternal stress and boosting emotional intelligence to support successful breastfeeding in mothers of preterm infants. Healthcare providers and administrators are encouraged to offer assistance and educational programs to enhance the emotional intelligence of mothers with preterm infants. Further scholarly investigation and targeted interventions in these areas are warranted to advance the overall well-being of both mothers and infants in the neonatal intensive care unit.

## Data Availability

The datasets used and/or analyzed in the current study are available through the corresponding author upon reasonable request. The data are not publicly available due to restrictions, e.g., their containing information that could compromise the privacy of research participants.
